# Anticancer Potential of Resveratrol, β-Lapachone and Their Analogues

**DOI:** 10.3390/molecules25040893

**Published:** 2020-02-18

**Authors:** Danielly C. Ferraz da Costa, Luciana Pereira Rangel, Mafalda Maria Duarte da Cunha Martins-Dinis, Giulia Diniz da Silva Ferretti, Vitor F. Ferreira, Jerson L. Silva

**Affiliations:** 1 Departamento de Nutrição Básica e Experimental, Instituto de Nutrição, Universidade do Estado do Rio de Janeiro, Rio de Janeiro 20550-013, Brazil; danielly.costa@uerj.br; 2 Faculdade de Farmácia, Universidade Federal do Rio de Janeiro, Rio de Janeiro 21941-902, Brazil; lprangel@pharma.ufrj.br; 3 Programa de Biologia Estrutural, Instituto de Bioquímica Médica Leopoldo de Meis, Instituto Nacional de Ciência e Tecnologia de Biologia Estrutural e Bioimagem, Universidade Federal do Rio de Janeiro, Rio de Janeiro 21941-902, Brazil; mafaldamariamartins@gmail.com (M.M.D.d.C.M.-D.); giuliadiniz@hotmail.com (G.D.d.S.F.); 4 Departamento de Tecnologia Farmacêutica, Faculdade de Farmácia, Universidade Federal Fluminense, Rio de Janeiro 24241-000, Brazil; vitorferreira@id.uff.br

**Keywords:** resveratrol, β-lapachone, cancer

## Abstract

This review aims to explore the potential of resveratrol, a polyphenol stilbene, and beta-lapachone, a naphthoquinone, as well as their derivatives, in the development of new drug candidates for cancer. A brief history of these compounds is reviewed along with their potential effects and mechanisms of action and the most recent attempts to improve their bioavailability and potency against different types of cancer.

## 1. Introduction

**Figure 1:**
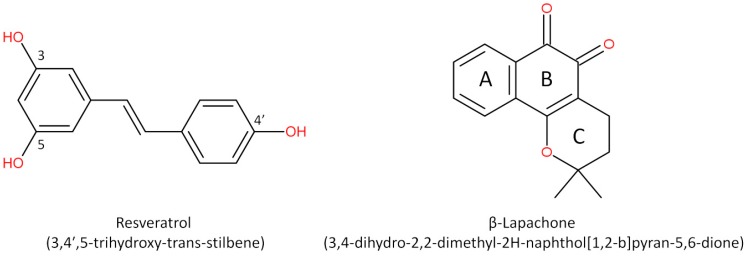
Structures of resveratrol and β-lapachone.

Cancer is a critical public health problem worldwide, with more than 18 million new cases and 9.6 million deaths estimated in 2018 [1]. Cancer therapeutics involves multiple combined approaches and requires the development of strategies based on the design and synthesis of promising compounds to improve treatment response. The search for new molecules with antitumor activity is still necessary and pursued by the pharmaceutical industry and many different research groups. Natural compounds have long been used for this purpose, leading to the development of new drugs, or as templates for new molecules with similar structures and effects [2]. Historically, bioactive compounds derived from animals and plants have been extensively used to treat diseases, which explain the scientific interest in natural products for drug discovery [3]. Both resveratrol and β-lapachone have been used with this purpose, leading to the development of new derivatives and the production of delivery systems aimed at improving the bioavailability of these compounds. In this review, we describe the potential of resveratrol, a polyphenol stilbene, and β-lapachone, a napthoquinone (Figure 1), as well as their derivatives, in the development of new drug candidates for cancer. We begin with a brief history of these compounds and move further to the discussion of their potential effects and mechanisms of action and the most recent attempts to improve their bioavailability and potency against different types of cancer.

## 2. Resveratrol and Stilbene-Based Compounds

Stilbenes are phytochemicals with small molecular weights (approximately 200–300 g/mol) found in a wide range of plants and dietary supplements [4]. Stilbene-based compounds have become of particular interest because of their wide range of biological activities. Among them, resveratrol (3,4′,5-trihydroxy-*trans*-stilbene) is a natural nonflavonoid polyphenol, classified as a phytoalexin, which can be naturally produced by more than 70 plant species (including grapes, blueberries, raspberries, mulberries and peanuts) in response to stressful conditions, such as fungal infection and ultraviolet radiation. In plants, the molecule exists as *trans*-resveratrol and *cis*-resveratrol isomers, and their glucosides, *trans*-piceid and *cis*-piceid. Since resveratrol is efficiently extracted from grape skin during the wine-making process, red wine is the most important dietary source of this bioactive compound [5,6,7]. Resveratrol has attracted scientific attention since 1997, when Jang et al. first demonstrated its ability to modulate in vivo carcinogenesis by inhibiting tumor initiation, promotion and progression in mice [8]. After that, the number of published papers regarding the role of resveratrol in blocking the multistep process of carcinogenesis increased substantially [9,10].

Currently, resveratrol is well characterized as a potent chemopreventive and chemotherapeutic agent in different cancer experimental models and clinical trials (for a review, see our recent publication) [11]. Several cell processes are targeted by this phytoalexin by upregulation or downregulation of multiple molecular pathways involved in cancer. Resveratrol modulates xenobiotic metabolism by inhibiting phase I cytochrome P450 enzymes responsible for carcinogen activation and by inducing phase II carcinogen detoxifying enzymes; reduces oxidative stress and inflammation by decreasing reactive oxygen species (ROS) generation and downregulating cyclooxygenase (COX) and inflammatory cytokines; promotes cell proliferation arrest by modulating cell cycle regulatory machinery such as cyclins and cyclin-dependent kinases (CDKs); induces apoptosis of damaged or transformed cells by different mechanisms, including upregulation of the p53 tumor suppressor protein and BAX and downregulation of Bcl2 and survivin; suppresses angiogenesis, invasion and metastasis by inhibiting hypoxia-induced factor-1α (HIF-1α) and matrix metalloproteinases; targets hormone signaling due to its relevant antiestrogenic activity in hormone-dependent cancers; and reduces the risk of multidrug resistance (MDR) via multiple targets related to carcinogenesis and chemo/radioresistance [12,13]. Resveratrol has also been proposed as a pro-oxidant agent depending on the concentration, exposure time and cell type. The oxidative damage caused by this compound represents one of the cytotoxic mechanisms involved in tumor cell death [14,15].

**Figure 2:**
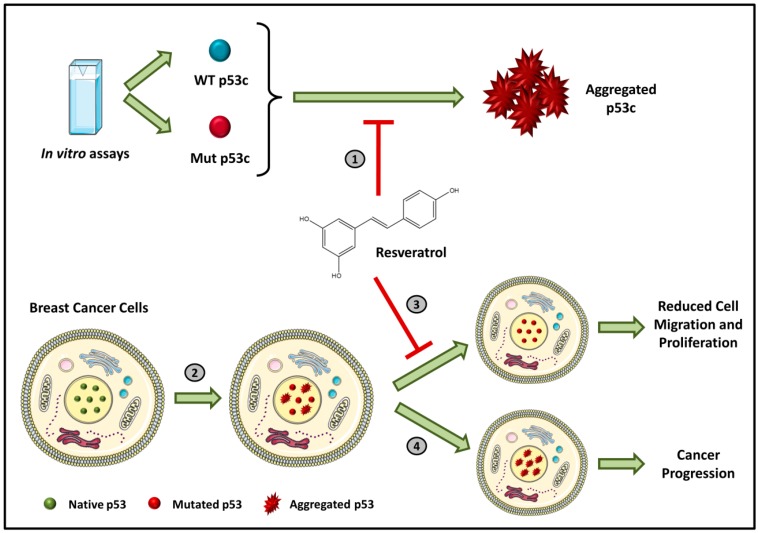
Schematic representation of p53 inhibition by resveratrol. In (**1**), the resveratrol in vitro capacity of inhibition of both WT and mutant p53 aggregation is described. (**2**) When mutations in the TP53 gene appear, the protein produced is less stable and forms aggregates. These aggregates are related to a direct effect in cancer proliferation and migration that is inhibited by treatment with resveratrol (**3**). Otherwise, cancer progression occurs (**4**). Extracted from Ferraz da Costa, 2018 [21].

Mutant p53 is associated with aggregation, which results in negative dominance and gain-of-function effects [16,17,18,19]. Novel compounds can directly target the interaction of p53 mutant aggregates with their p63 and p73 paralogues and with other transcription factors [17,20]. New compounds capable of intervening in the formation of aggregates can range from natural molecules such as resveratrol analogues, synthetic molecules such as Michael acceptors, small synthetic peptides, aptamers of nucleic acids and glycosaminoglycans [17]. These new drugs have great potential to represent radical innovation. In our recently published paper, we demonstrated that resveratrol inhibits the aggregation of p53 mutants *in vitro*, in tumor cells and in xenotransplants implanted in nude mouse models (Figure 2) [21]. However, very high doses were required to exert the effect. We intend to use synthetic chemistry to produce resveratrol analogs with higher potency that can be used as pharmaceuticals.

In another study, we described that transient transfection of the wild-type p53 gene causes H1299 cells (null to p53) to become more sensitive and responsive to the pro-apoptotic properties of resveratrol, similar to what was observed in MCF-7 cells. It was proposed that resveratrol could be used therapeutically in combination with other methods of promoting p53 activity in cells, such as gene therapy using the wild-type (WT) p53 gene or chemicals that restore p53 function [22].

In mammalian experimental models, resveratrol is extensively metabolized and quickly eliminated, resulting in poor bioavailability. After oral administration, resveratrol is absorbed by passive diffusion or by membrane transporters at the intestinal level and is then released in the bloodstream, where it can be detected as an unmodified or metabolized molecule [13,23]. Although 75% of resveratrol is absorbed through the oral route, only 1% is detected in the blood plasma after the whole metabolism [24]. In recent years, different methodological approaches and synthetic derivatives have been developed to improve resveratrol bioavailability. Many studies have been performed to synthesize new and more effective resveratrol analogs that display better pharmacokinetic properties, low toxicity and minimum side effects. The methoxylated, hydroxylated and halogenated resveratrol derivatives are more explored due to their beneficial biological activities and increased oral bioavailability [25]. Previous studies showed that methoxylation increases metabolic stability and the time length required for the molecules to reach the plasma concentration peak. Moreover, the substitution of hydroxyl groups of resveratrol to methoxyl groups substantially potentiated its therapeutic versatility. It was also reported that the introduction of additional hydroxyl groups significantly increased the biological activity of resveratrol analogs [23,25]. In this review, we collect and present recent evidence in the literature regarding resveratrol derivatives and their anticancer effects, with an emphasis on the molecular mechanisms involved.

### 2.1. Methoxylated Resveratrol Derivatives

#### 2.1.1. Pterostilbene

Pterostilbene (*trans*-3,5-dimethoxy-40-hydroxystilbene) is a naturally occurring stilbene, found mainly in blueberries and grapes. It is a dimethylated derivative of resveratrol with comparable antioxidant, anti-inflammatory and anticarcinogenic properties [26]. Substituting its hydroxyl for a methoxyl group enhances the lipophilicity of pterostilbene, adding to its in vivo bioavailability and, thus, improving the biological activity of this compound compared to resveratrol [27]. A study designed to compare the bioavailability, pharmacokinetics and metabolism of resveratrol and pterostilbene following equimolar oral dosing administered in rats showed that resveratrol and pterostilbene were approximately 20% and 80% bioavailable, respectively [28]. Cumulative experimental data have noted that pterostilbene exerts multiple effects against a variety of cancer models through modulation of the cell cycle, induction of cell death, and inhibition of invasion and metastasis [26,29].

The first evidence of the anticancer properties of pterostilbene was demonstrated in a colon tumorigenesis model. Pterostilbene was shown to decrease the expression of inflammatory genes, such as iNOS in the colonic crypts and aberrant crypt foci (ACF) in rats, thus suggesting that its anti-inflammatory properties may be critical in colon cancer prevention [30]. Additionally, this compound inhibits preneoplastic lesions and adenomas in mouse colon by suppressing GSK3β phosphorylation and Wnt/β-catenin signaling pathway and reduces the expression of cyclin D1, vascular endothelial growth factor (VEGF) and matrix metalloproteinases (MMPs) [31]. It was also reported that pterostilbene showed significant dose-dependent antiproliferative and cytotoxic effects and inhibited Myc, beta-catenin and cyclin D gene expression in human colon cancer Caco-2 cells [32]. In gastric adenocarcinoma cells, pterostilbene inhibits cellular proliferation and leads cells to apoptosis by different pathways, such as caspase cascade activation and modulation of cell-cycle regulating proteins [33]. In a human model of hepatocellular carcinoma, pterostilbene suppresses tumor growth by interfering in the signal transduction pathways of NF-κB and on the expression of VEGF, matrix metalloproteinase-9 (MMP-9), AP-1 and mitogen-activated protein kinase (MAPK) [34,35]. Breast cancer stem cells isolated from MCF-7, which expresses the surface antigen CD44+/CD24–, were selectively eliminated by pterostilbene. Furthermore, this compound induces necrosis and inhibits mammosphere formation, increases the activity of paclitaxel, decreases CD44 expression, induces β-catenin phosphorylation through the inhibition of hedgehog/Akt/GSK3β signaling and decreases the expression of c-Myc and cyclin D1 [36]. More recent studies showed that pterostilbene is a promising agent against human papillomavirus (HPV) E6+ tumors tested in vitro and in vivo. In vitro, this compound downregulates the viral oncogene E6. On the other hand, in mouse TC1 tumors, in addition to inhibiting E6, pterostilbene suppressed VEGF and tumor development [37]. In acute lymphoblastic leukemia cell lines Jurkat and Molt-4, the potential of pterostilbene to modulate Fas, a member of the death-inducing family of tumor necrosis factor (TNF), was investigated. Pterostilbene increased both Fas mRNA and its cell surface levels, thus leading to apoptosis [38].

#### 2.1.2. Trimethoxystilbene

Trimethoxystilbene (*trans*-3,4′,5-trimethoxystilbene) is also a methylated resveratrol derivative, described as a potent chemopreventive agent, which promotes the induction of cell cycle arrest, reduces angiogenesis, inhibits cancer cell proliferation, increases apoptosis and decreases metastasis [25]. In MCF-7 breast cancer cells, this compound acts by inhibiting epithelial–mesenchymal transition (EMT), negatively modulating β-catenin nuclear translocation and phosphatidylinositol 3-kinase (PI3K)/protein kinase B (AKT) signaling [39]. Its anticancer mechanisms on lung cancer cells involve apoptosis induction by activation of caspases 3 and 9 and poly (ADP-ribose) polymerase interruption [40]. Additionally, in human lung cancer (A549), trimethoxystilbene promotes a decrease in invasive, migratory and adhesive characteristics of these cells and modulates the mRNA levels that encode for MMP-2 protein [41]. When evaluating the effect of this resveratrol derivative in rat C6 and human T98G glioma cells, a massive accumulation of cells at the G2/M phase of the cell cycle and apoptosis via caspase-3 related to p53 tumor suppressor protein induction were observed [42]. Trimethoxystilbene is a more effective derivative than resveratrol in suppressing the growth of HepG2 hepatocellular carcinoma cells via induction of G2/M cell cycle arrest (by upregulation of cyclin B1) and apoptosis (by downregulation of Bcl-2) [43].

#### 2.1.3. Tetramethoxystilbene

The modification of the resveratrol structure that generates its analogue tetramethoxystilbene improved its bioactivity by suppressing cell growth in prostate, colon, ovarian and hepatocellular cancer cells. This analogue demonstrates a higher activity on human melanoma A375 by decreasing cell proliferation after treatment using a lower dose (IC_50_ = 0.7 µM) than resveratrol (IC_50_ = 100 µM) [44]. Compared with the *trans* form of the 3,4,5,4′-tetramethoxystilbene resveratrol derivative compound, the *cis* form is ten times more potent at decreasing the growth of human WI38VA virally transformed fibroblasts [45]. Using a xenograft of human ovarian cancer (A2780 and SKOV-3) as a model to study the effect of *trans*-3,4,5,4′-tetramethoxystilbene, it was observed that treatment with this derivative is able to reduce tumor cell growth [46,47]. For breast cancer, it was demonstrated that proapoptotic proteins and voltage-dependent anion channel 1 (VDAC-1) expression were increased after treatment [48]. As a new approach to treat osteosarcoma cells resistant to paclitaxel and cisplatin, the use of tetramethoxystilbene decreases, in vitro and in vivo, the viability of resistant cells and induces massive apoptosis [49].

#### 2.1.4. Pentamethoxystilbene

Pentamethoxystilbene is a hybrid molecule chemically synthesized and, similar to resveratrol, has low oral bioavailability but presents high intravenous bioavailability. In breast carcinoma cells (MCF-7), this derivative is a good antiproliferative candidate that acts through different pathways as a G1 cell cycle regulator, modulating cyclins E and D and retinoblastoma protein (pRb). It is a better suppressor agent for breast cancer cells than resveratrol or other methoxylated derivatives. At the IC_50_ concentration of this derivative (37.8 µM), treatment with resveratrol only reduces cell survival 20% [50]. In colon cancer, a better response was reached with this compound, with activation of apoptosis through cell cycle arrest in G2/M phase, polymerization of microtubules and finally caspase-induced apoptosis. Beyond apoptosis, the compound may decrease iNOS, β-catenin and cell proliferation [51,52]. No further studies have been published with this derivative since 2012.

### 2.2. Hydroxylated Resveratrol Derivatives

#### 2.2.1. Dihydroxystilbene

The resveratrol analogue 4,4′-dihydroxy-*trans*-stilbene (4,4′-DHS) was designed to improve resveratrol efficiency, both as an antioxidant and antiproliferative agent. 4,4′-DHS exhibits remarkably higher cytotoxicity than resveratrol against human promyelocytic leukemia (HL-60) cells [53]. 4,4′-DHS also inhibits the clonogenic efficiency of fibroblasts nine times more potently than resveratrol, although with a different mechanism. 4,4′-DHS predominantly induces an accumulation of cells in G1 phase, whereas resveratrol disturbs the G1/S phase transition. Furthermore, 4,4′-DHS increases p21 and p53 protein levels, whereas resveratrol leads to phosphorylation of the S-phase checkpoint protein Chk1 [54]. In a mouse lung cancer model, 2,3- and 4,4′-dihydroxystilbene (at 10 and 25 mg/kg, administered twice daily) inhibited tumor growth and metastasis. The antitumor and antimetastatic effects of these compounds were partly due to anti-lymphangiogenesis and the regulation of M2 macrophage activation and differentiation [55]. The cytotoxic action of 4,4′-DHS was also investigated in vitro in human neuroblastoma cell lines and in a mouse xenograft model of human neuroblastoma. The pharmacological action of 4,4′-DHS in the human neuroblastoma IMR32 cells was mediated by the destabilization of mitochondrial and lysosomal membranes, associated with modulation of several related pro- and anti-apoptotic cascades of proteins. Additionally, in the animal model, the oral administration of 4,4′-DHS for one month was well tolerated and demonstrated a greater therapeutic potential than resveratrol [56]. More recently, Saha et al. demonstrated that in melanoma cells, 4,4′-DHS acts by inducing apoptosis and cell cycle arrest in G1 phase and inhibiting cell proliferation. A significant reduction of melanoma tumors in a preclinical murine model was observed, and the antimetastatic effect of 4,4′-DHS was shown in a melanoma-mediated lung metastasis model in vivo [57]. In vivo assays performed in different mouse models of tumor xenografts demonstrated that 4,4′-DHS was able to disrupt the DNA replication pathway, leading to the apoptosis of pancreatic, ovarian and colorectal cancer cells [58].

#### 2.2.2. Tetrahydroxystilbene

Tetrahydroxystilbene is a natural resveratrol analogue with multiple biological activities. In SK-Mel-28 melanoma cells, treatment with this compound induced apoptosis and inhibited cell proliferation [59,60]. In prostate cancer, its anticancer mechanisms involve JAK1 leading to STAT3 activation, which leads to a cytokine signal transduction pathway [61]. In liver and colon cancer cells, tetrahydroxystilbene arrests the cell cycle at G1 phase by modulating the cyclins pathway [62,63]. In human leukemia cells (U937), this compound induces massive apoptosis and leads to cell cycle arrest in G1 phase by regulating Bcl-2 and cIAP-2 (anti-apoptotic proteins). In cervix cancer, it acts by modulating p53 protein, thus leading cells to apoptosis [64,65]. In the last year, the 2,3,5,4′-tetrahydroxystilbene-2-O-β-D-glucoside (THSG) derivative was the major compound studied. In HT-29 colon adenocarcinoma cells, treatment with this compound reduced cell migration, invasion and adhesion, thus inhibiting metastasis. This is possible because THSG inhibits NF-κB pathway activation and consequently suppresses proteins involved in migration and invasion, such as MMP-2 and p-VE-cadherin, and ICAM-1 proteins involved in cell adhesion [66]. In vivo assays with THSG, using azoxymethane-induced colorectal cancer in rats, induced a 50% reduction in total colonic aberrant crypt foci by the inhibition of NF-κB pathway activation [67]. In MCF-7 breast cancer cells exposed to adriamycin and THSG, the vascular endothelial growth factor/phosphatidylinositol 3-kinase/Akt pathway was inhibited, triggering apoptosis by modulating Bcl-2 and caspase-3 [68].

#### 2.2.3. Hexahydroxystilbene

Hexahydroxystilbene is a synthetic resveratrol derivative with higher biological activity [69]. When tested in breast cancer cells, hexahydroxystilbene induced apoptosis and inhibited cell proliferation by p53 protein accumulation and downregulation of mitochondrial superoxide dismutase [70]. In human colon cancer cells (HT-29), treatment with this compound leads to apoptosis and cell cycle arrest [71]. Similar results were observed in leukemia cells, with apoptosis induction by caspase activation pathways [72]. In vivo assays in a melanoma mouse model also demonstrate the induction of apoptosis pathway by upregulation of p21, downregulation of CDK-2 and cell cycle arrest at the G2/M phase [73]. Hexahydroxystilbene demonstrates an antiproliferative effect and accelerates senescence in cultured human peritoneal mesothelial cells by an oxidative stress-dependent mechanism. Treatment with 10 µM hexahydroxystilbene promoted an increase in 8-OHdG levels, a product of DNA oxidation, and a time-dependent increase in ROS release was also reported. On the other hand, soluble factors released by human peritoneal mesothelial cells that senesced prematurely in response to treatment promoted the growth of colorectal and pancreatic carcinomas in vitro [74]. No further studies have used this compound since 2013.

Although many studies have reported that stilbene compounds could play essential roles as chemotherapeutic agents by regulating multiple mechanisms and acting on different targets, further translational research is required to determine if the preclinical anticancer properties of these compounds, either alone or as part of combined therapies, are applicable in a clinical setting. The IC_50_ values for resveratrol and its derivatives vary in a wide range, as indicated in Table 1.

## 3. β-Lapachone and Its Derivatives: The South American Promise for Cancer

Quinones are widely distributed in nature, products of the secondary metabolism of several different species, with an incredible variety of biological responses [85,86]. Quinones may act as vitamins, antioxidants and are capable of stimulating antibacterial, antiallergic and anticancer effects, among others [87], which motivate their investigation as a therapeutic tool. Quinones can be cytotoxic through several mechanisms of action, including redox cycles, arylation of the thiol groups of proteins, intercalation, induction of breaks in the DNA chain, generation of free radicals and other ROS and bioreductive alkylation of critical cellular proteins and DNA via the formation of quinone methide. Besides basic research studies, quinone-based compounds are already used in the clinic. For instance, in cancer, doxorubicin, mitoxantrone and mitomycin C are used, among others [88,89]. Among all naphthoquinones described to date, three of them are widely known, mostly due to their anticancer effects and the story of their discovery—lapachol, β-lapachone and α-lapachone—of which lapachol (2-hydroxy-3-(3-methyl-2-butenyl-)-1,4-naphthoquinone, C_15_H_14_O_3_), with a molecular weight of 242.2738 g/mol, was isolated first from the heartwood of *Tabebuia impetiginosa*, a widespread tree species in Brazil and other South American countries [90]. No antitumor properties were reported for lapachol until Rao et al., in 1962, described a potent anticancer effect in rats [91]. Since then, a great interest in the research of the anticancer properties of lapachol and its derivatives, or structural isomers, α- and β-lapachone, has risen [92].

β-lapachone (3,4-dihydro-2,2-dimethyl-2H-naphthol[1,2-b]pyran-5,6-dione, C_15_H_14_O_3_), molecular weight 242.2738 g/mol, is an isomer of lapachol and has been described to promote several biological effects, such as anti-inflammatory, antibacterial and anti-*Trypanosoma* [93,94], and most important for this review, anticancer properties in different cancer types such as pancreatic cancer [95], breast cancer [96], hepatocellular carcinoma [97] and others. Several derivatives have been developed throughout the years, and it is noteworthy that a β-lapachone pro-drug, with commercial name ARQ-761, is in phase I/II of clinical studies for solid tumors [98].

### 3.1. Anticancer Effects

As mentioned previously, there is a plethora of studies that demonstrate the ability of β-lapachone to induce cell death in several cancer cell lines, [95,96,97,99,100,101], but depending on the type of cancer, it is able to induce different types of cell death. Many studies demonstrate that β-lapachone is capable of inducing apoptosis [96,101,102] in cells such as HepG2, a hepatocellular carcinoma cell line [103], but, on the other hand, others demonstrate an ability to induce cell death via necroptosis, which is a type of organized necrosis [104,105,106]. As another example, Park et al., 2014 [97], showed that β-lapachone is capable of inducing this type of cell death in SK-Hep1, another hepatocellular carcinoma cell line.

Most anti-neoplastic drugs demonstrate a cytostatic effect, meaning that they are able to inhibit cell proliferation, and the ability of β-lapachone to prevent the proliferation of cancer cells has long been described [107]. IC_50_ values vary in a wide range, depending on the model tested (Table 2). As observed for the type of cell death that is induced by β-lapachone, the mode of cell cycle arrest is also dependent on the cell type under study. Dias et al. (2018) demonstrated that lapachone and its iodine derivatives induce cell cycle arrest in G2/M in human oral squamous cell carcinoma cells, and Lai et al. (1998) [108] showed cell cycle arrest in the S phase for a hepatoma cell line (HepA2).

There is also evidence of antitumoral effects of β-lapachone in preclinical studies. Wu et al. reported the promotion of heat shock protein 90 cleavage by β-lapachone, mediated by oxidative stress in NQO1-expressing cell lines. In the same work, in a mouse xenotransplant model, human lung cancer xenograft growth and angiogenesis were inhibited by β-lapachone treatment [109]. Kee et al. also demonstrated that β-lapachone is able to suppress lung metastasis of melanoma in an experimental mouse model [102].

### 3.2. Mechanisms of Action

#### 3.2.1. ROS and NQO1

The primary mechanism of action of β-lapachone and its derivatives is the formation of ROS [92] through its processing by NAD(P)H quinone oxidoreductase 1 (NQO1). This enzyme is able to catalyze a futile redox cycle, leading to the formation of unstable hydroquinone, which is rapidly oxidized back to the original quinone under aerobic conditions [114]. The continuous redox cycles eventually oxidize a large number of reduced pyridine nucleotides, which form ROS [115]. This effect is quite robust, since one mol of β-lapachone is capable of generating 120 mol of superoxide in two min, consuming 60 mol of NAD(P)H [106], which results in a rapid depletion of intracellular NAD+ pool over 20 to 30 min [116]. This abnormal production of ROS leads to an increase in Ca^++^, depolarization of the mitochondrial membrane and a decrease in ATP synthesis. Therefore, in a general way, the activation of β-lapachone by NQO1 leads to cell death by apoptosis [117,118]. There are several studies that show that β-lapachone leads to the formation of ROS in cancer cells, such as Park et al., in 2014, who report that the increase of ROS is capable of inducing cell death of hepatocellular carcinoma cells (SK-Hep1) [97]. In 2011, the same group showed that ROS were involved in β-lapachone-induced autophagy in glioma cells (U87 MG). Bey et al. (2013) showed that H_2_O_2_ is the primary obligate ROS species necessary for β-lapachone breast cancer cell cytotoxicity through lipid peroxidation, which damages cellular membranes and organelles [106].

It is important to note that several types of solid tumors, such as cholangiocarcinoma [119], lung [120], pancreas [121], breast [122] and squamous cell carcinoma of the uterine cervix [123], have high expression of NQO1, and there are studies that demonstrate a β-lapachone preferential tropism for NQO1-positive cells [124].

Liver tumors are a very interesting case, since normal hepatocytes do not express NQO1 [125,126], but preneoplastic lesions and hepatic tumors demonstrate the presence of this enzyme [127,128]. Thus, this differentiated expression may be very important in the development of targeted therapies since it induces cell death of neoplastic or preneoplastic cells, greatly reducing side effects on healthy liver cells. Additionally, hyperthermia has been reported as an enhancer of β-lapachone effects due to the increase in NQO1 levels after heat shock and its stabilization by HSP70 [129,130,131,132]. Finally, it is noteworthy that NQO1-positive breast cancer cells correlate with the malignancy of the disease and could be used as a prognostic biomarker for breast cancer [124].

#### 3.2.2. Topoisomerase Inhibition

One of the first mechanisms of action reported for β-lapachone was its role as a topoisomerase I modulator. Earlier thought of as a topoisomerase activator [133,134,135], it was later shown to act as an inhibitor, through the demonstration that β-lapachone inhibits the catalytic activity of topoisomerase I (purified from calf thymus and human cells), through its direct binding, since the incubation of topoisomerase I with β-lapachone (before adding DNA) considerably increased its inhibition, but the incubation of topoisomerase I with DNA prior to the treatment did not show any effect [136]. Other studies refer to similar effects in cell lines of different cancers, such as prostate cancer, breast cancer and leukemia [96,137,138].

#### 3.2.3. p53

A number of studies have demonstrated β-lapachone effects on p53 independently of the cell p53 status (no expression or expression of wild-type or mutant p53); in all cases, cells can be sensitive to the effects of β-lapachone [107,139,140]. Huang Pardee, in 1999 [107], reported that β-lapachone had the ability to drastically reduce levels of mutant p53 in colon cancer cells, although it did not alter the expression levels of wild-type p53. In addition, p53 can also be regulated by β-lapachone through its phosphorylation and consequent activation, with no modification in its expression levels [141]. Finally, Pink et al. (2000) reported the β-lapachone-mediated activation of a cysteine-protease capable of digesting several cellular proteins, including p53 [142].

#### 3.2.4. Other Cellular or Molecular Pathways

Yu et al. demonstrated the anti-tumor effect of β-lapachone on breast cancer tumors with a variation in the phosphorylation of AKT, 4EBP-1 and S6, which are related to the mTOR pathway and is also related to the apoptotic activity of this compound in gastric carcinoma cells [101]. Additionally, Wu et al., in 2016 [109], demonstrated that the effect of β-lapachone on reducing the growth of lung cancer cell tumors is related to AKT. In addition to the mTOR pathway, E-cadherin was altered in tumors, and Kim et al. (2007) [102] suggested that β-lapachone inhibits the progression and metastasis of hepatocellular carcinoma by increasing the expression of this protein and other proteins of the mTOR pathway. Additionally, the inactivation of the Akt/mTOR pathway was again attributed to β-lapachone, promoting the inhibition of EMT transition in NQO1-positive cells.

### 3.3. Strategies to Overcome β-Lapachone Bioavailability and Toxicity Issues: Drug Delivery and Derivatives Synthesis

Even though β-lapachone is a promising anticancer drug, its low bioavailability represents a limitation for clinical use due to low solubility in water and gastrointestinal fluids [143]. There is also a concern with the low concentration reached in the target cells and systemic toxicity, since β-lapachone displays a general distribution pattern and a dose limitation because of the risk of methemoglobinemia through the generation of nonspecific ROS at high doses [116,143].

As mentioned before, β-lapachone (Table 2) has a molecular weight of 242.29 g/mol; it is a small molecule, nonionized in the intestinal system, with pH-independent solubility. The experimental solubility value is very similar to the theoretical value of 48.33 µg/mL [112,144]. β-lapachone has the potential to be orally administered; its estimated oral fraction absorbed and intestinal effective permeability value is 85% [145]; however, a bioavailability of 15.5% through oral administration was shown in rats, probably due to broad metabolic first-pass degradation in the liver, intestines and low solubility in water, with a slow dissolution rate in the intestinal tissue [144]. Moreover, preclinical studies demonstrate that, to promote a better absorption of β-lapachone, different formulations would be necessary, and due to these findings, along with its first-pass metabolism, it was considered a difficult drug for oral administration [144,146].

Due to the low availability and unspecific toxicity of β-lapachone, there is a constant need for the development of new drug delivery systems to increase bioavailability to promote its use. There are two classical systems of β-lapachone delivery, cyclodextrin inclusion complexes and liposomes, although most studies are concerned with physical–chemical and absorption properties without any cancer therapy approach. A thorough review on this subject was written by Ferreira et al., 2016 [147]. Here, we focus on anticancer approaches.

The intricate structure of cyclodextrin allows the formation of β-lapachone/cyclodextrin inclusion complexes because of its hydrophobic core [145,148]. These inclusion complexes are capable of increasing drug bioavailability, altering their permeability, dissolution properties or both [149]. Seoane et al. (2013) used a methylated-β-cyclodextrin/poloxamer 407 mixture to create a delivery system of β-lapachone and evaluated its antitumoral activity. This system has the particularity of forming a gel above 29 °C, which facilitates intratumoral and extended drug delivery. MCF-7 tumor-bearing mice treated with this system, by intratumoral injection, showed a reduction of tumor volume without apparent liver and kidney toxicity [150].

Liposomes (one or multiple layers of phospholipids) are very interesting delivery systems because they can be used for both hydrophobic and hydrophilic drugs and display remote drug loading, homogeneous particle size, long-circulating stability, specific release and the ability to lower drug toxicity [151]. Liposomes with β-lapachone, in different mixed micellar formulations of phosphatidylcholine, sodium deoxycholate and sodium lauryl sulfate (SLS), showed the ability to increase gastrointestinal absorption at different sites (particularly in the large intestines) due to β-lapachone solubilization and interaction with the intestinal membrane [152].

Recently, there has been an increased exploration of nanosystems or nanoparticle delivery systems to surpass the limitations of these delivery systems and increase specificity and cytotoxicity to cancer cells. Dai et al. (2019) [153] developed a nanosystem with a charge-reversal ability and self-amplifiable drug release system that encapsulated β-lapachone in a pH/ROS cascade-responsive polymeric prodrug micelle. They showed that this system is capable of effectively increasing cell uptake and specific delivery through acidity-activating charge conversion and ROS-response drug release. Upon the uptake of β-lapachone, ROS formation was increased (NQO1-mediated), which lowered the cell ATP levels and consequently reduced P-gp-mediated drug efflux, decreasing multidrug resistance (MDR). This caused a massive reduction of MCF-7 tumors treated with this β-lapachone delivery system and low systemic toxicity [153].

Another strategy to increase the therapeutic efficacy of β-lapachone, through the reduction of MDR, is to promote its codelivery with another drug, such as doxorubicin. This was an option used by Li et al., demonstrating that a nanostructured lipid carrier (NLC) codelivering β-lapachone and doxorubicin had a higher therapeutic efficacy in breast cancer tumor-bearing mice, leading the authors to propose this as a possible strategy to overcome MDR in breast cancer [154].

A nanoparticle developed by Yin et al., a ROS-responsive block copolymer prodrug that self-assembles into polymeric micelles that encapsulate β-lapachone, increased tumor-specific ROS formation after intravenous administration in tumor-bearing mice. This increased ROS level and triggered drug release, allowing maximization of therapeutic efficacy, suppression of tumor growth and minimization of systemic toxicity [155].

There are three main strategies to develop β-lapachone derivatives or analogues—A- and C-ring modifications and redox center modifications—which can be obtained by copper-catalyzed azide-alkalyne cycloaddition, palladium-catalyzed cross couplings and heterocyclization reactions [156]. The great progress seen in this field is due to essential contributions from Brazilian research groups, such as Vitor Francisco Ferreira and Eufrânio Nunes da Silva Júnior [156,157,158,159,160,161]. However, most studies of β-lapachone derivatives or analogues are composed of a series of compounds with different modifications and analysis of the possible anticancer effect in cancer cell lines [89,156,161]. Nevertheless, there are more in-depth studies that attempt to understand the mechanisms of action of the compounds and eventually test them on experimental tumor models.

Recently, Dias et al. (2018) [113] showed that β-lapachone and its 3-iodine derivatives (3-I-α-lapachone and 3-I-β-lapachone) were able to induce significant cytotoxicity against different types of cancer cells (Table 2), with cell cycle arrest in G2/M, DNA fragmentation, increase in apoptosis protein levels and morphology, and production of ROS. This work also demonstrated that these compounds were able to reduce tumor burden for mice xenotransplanted with breast adenocarcinoma cells, without any alteration of biochemical, hematological or histological parameters of the treated mice, showing a nonsignificant systemic toxicity. Li et al. described a new class of β-lapachone derivatives, naphtho[2,1-d]oxazole-4,5-diones (Table 2), with higher solubility and comparable activities, both in vitro and in vivo (xenotransplants), against NQO1-positive A549 lung cancer cells [112]. The compounds β-lap-dC3 and -dC6 are prodrug diester derivatives of β-lapachone. When encapsulated in PEG-β-PLA micelles, they were described to be more efficient in loading micelles than β-lapachone itself, and β-lap-dC3 improved the survival rate of NSCLC xenograft-bearing mice, increasing β-lapachone concentration in the target tissues, which makes it a promising therapy to be developed against NQO1-positive cells [162].

Finally, the sensitization of cells promoted by different energy sources, such as light or radiation, in combination with β-lapachone and its derivatives has also been described. Lamberti et al. (2018) showed the synergism between halogenated derivatives of β-lapachone and photodynamic therapy in melanoma cells with positive results due to the upregulation of NQO1 expression [163]. Ionizing radiation at low doses was applied in combination with sublethal doses of β-lapachone in non-small-cell lung cancer (NSCLC) cell lines and in xenograft models in vivo [164]. Additionally, the massive release of ROS promoted by ionizing radiation was applied together with nontoxic β-lapachone doses to head and neck patient samples [165]. These results suggested the use of this combination to increase the efficacy of radiotherapy in NQO1-positive tumors and shall be tested in clinical trials in the near future.

## 4. Conclusions

Both of the molecules reviewed here, β-lapachone and resveratrol, are paradoxical. While they are naturally occurring products, or at least derived from natural sources, they can be radically improved by human manipulation. They represent a case study for natural products that can be enhanced by structural manipulation and/or delivery systems. In this paper, we intended to compile the available information about β-lapachone and resveratrol, with a special focus on what has been found in recent years and on the possible impact in cancer therapy, also showing how a bioactive compound/molecule can be found in natural resources, which are a possible reservoir of new therapies. However, it is essential not to be dismissive of the importance of manipulating natural molecules to increase their efficiency and specificity. The results obtained with β-lapachone, resveratrol and their analogues for cancer therapy are promising, especially if we manage to improve their specificity for cancer cells, with less systemic toxicity (a major problem for most chemotherapies) and their delivery, particularly by making them orally bioavailable for patient comfort. The similarities and differences between the two groups of compounds can be observed through the comparison between the values shown in Table 1 and Table 2 and the properties listed in the supplementary material tables (Tables S1–S4).

## Figures and Tables

**Table 1:** IC_50_ values of resveratrol and derivatives in different study models.

Compound	2D Structure	IC_50_	Study Model	References
**Resveratrol and Resveratrol Methoxylated Derivatives**				
Resveratrol	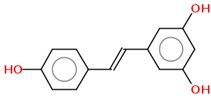	15–145 µM	Breast (MCF-7, MDA-MB-231, MDA-MB-468, MDA-MB-453), lung (A549, H460), pancreatic (Colo-357, Panc-1), prostate (LNCap, DU145) and colon (HCT116, Caco2) cancer cells; cervix carcinoma (HeLa); hepatocarcinoma (HepG2); melanoma (A357, SK-MEL-31); glioma (C6, T98G)	[22,42,75,76,77,78]
Pterostilbene	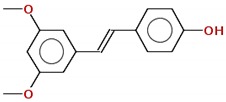	4.1–108 µM	Acute lymphoblastic leukemia (Molt-4), acute T cell leukemia (Jurkat), breast (MDA-MB-231), colon (COLO 205, HCT-116, HT-29) and endometrial (HEC-1A, ECC-1) cancer cells; melanoma (A357); hepatocarcinoma (HepG2); cervix carcinoma (HeLa, SiHa); epidermoid carcinoma (CaSki);	[27,38,79,80,81]
Trimethoxystilbene	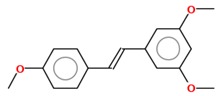	0.08–80.3 µM	Colon (Caco2, SW480), head and neck (KB), lymphoma (TK6) and breast (MCF-7) cancer cells; glioma (C6, T98G)	[39,42,82]
Tetramethoxystilbene	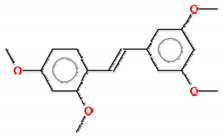	4-60–µM	Leukemia (HL-60) and breast (BT-459) cancer cells, melanoma (SK-MEL); cervix carcinoma (HeLa)	[83]
Pentamethoxystilbene	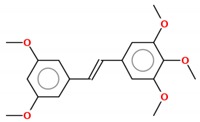	29.2–37.8 µM	Breast (MCF-7) and colon (Colon26) cancer cells	[50,52]
**Hydroxylated Resveratrol Derivatives**				
Dihydroxystilbene	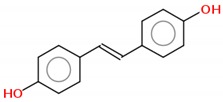	2.3–6.5 µM	Leukemia (HL-60), colon (HCT-116) and breast (MDA-MB-231) cancer cells; osteosarcoma (U2OS)	[57]
Tetrahydroxystilbene	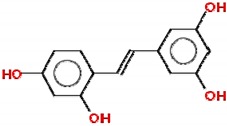	58.4–620.6 µM	Acute T cell leukemia (Jurkat), breast (MCF-7), lung (H1299, A549) and prostate (LNC) cancer cells;	[68,72,84]
Hexahydroxystilbene	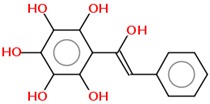	6.25–127.8 µM	Breast (T47D, ZR-75-1, MDA-MB-231), colon (HT-29), leukemia (HL-60) cancer cells; melanoma (M24met)	[69,70,71,73]

**Table 2:** IC_50_ values of β-lapachone and derivatives in different study models.

Compound	2D Structure	IC_50_	Study Model	References
Lapachol	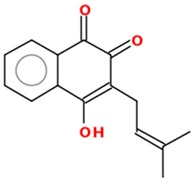	16.04–72.3 µM	Human chronic myelogenous leukemia (K562, Lucena), Burkitt’s lymphoma (Daudi), Breast cancer (MCF-7, SK-BR3)	[110,111]
ß-lapachone	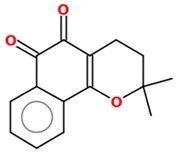	0.03–70.13 µM	Lung cancer cells (A549 cell line); Tongue squamous cell carcinoma (HSC-3, SCC4, SCC9, SCC15, SCC25), hepatocellular carcinoma (HEPG2), HL-60, K562, Gastric adenocarcinoma (AGP-01, ACP-02, ACP-03), colon adenocarcinoma (HT-29, HCT-116).	[112,113]
α-lapachone	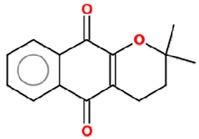	38–69 µM	K562, Lucena, Daudi, MCF-7	[111]
3-iodo-ß-lapachone	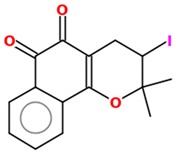	0.02–5.61 µM	Tongue squamous cell carcinoma (HSC-3, SCC4, SCC9, SCC15, SCC25), hepatocellular carcinoma (HEPG2), HL-60, K562, Gastric adenocarcinoma (AGP-01, ACP-02, ACP-03), colon adenocarcinoma (HT-29, HCT-116).	[113]
3-I-α-lapachone	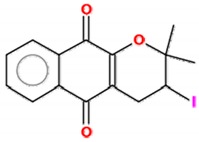	0.77–14.65 µM		
naphtho[2,1-d]oxazole-4,5-diones	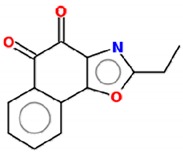	4.6–20 µM *	Lung cancer cells (A549 cell line)	[112]

* IC_50_ values range for all derivatives shown in reference [112].

## Supplementary Materials

The following are available online, Table S1: Bioactivity prediction (druglikeness*) of β-lapachone and its derivatives for specific biological activities; Table S2: Bioactivity prediction (druglikeness*) of resveratrol and its derivatives for specific biological activities; Table S3: Chemical and pharmacological properties of Lapachol, β-lapachone and derivatives; Table S4: Chemical and pharmacological properties of resveratrol and derivatives.
