# Supplementary material for: Anticancer Potential of Resveratrol, β-Lapachone and Their Analogues
Source: Molecules. 2020 Feb 18;25(4):893. doi: 10.3390/molecules25040893 (PMC7070981; doi:10.3390/molecules25040893)
Supplement: Supplementary file 1 [file molecules-25-00893-s001.pdf]

Supplementary Table 1. Bioactivity prediction (druglikeness*) of β-lapachone and its derivatives for specific biological activities

Compound	GPCR ligand	Ion channel modulator	Kinase inhibitor	Nuclear receptor ligand	Protease inhibitor	Enzyme inhibitor
**Lapachol**	-0.17	0.27	-0.11	0.20	-0.09	0.65
**ß-lapachone**	-0.08	0.33	-0.25	0.07	0.08	0.45
**α-lapachone**	-0.24	-0.12	-0.29	0.14	-0.18	0.26
**3-iodo-ß-lapachone**	-0.13	0.20	-0.21	0.03	0.01	0.50
**3-I-α-lapachone**	-0.28	-0.24	-0.24	0.09	-0.24	0.32
**naphtho[2,1-d]oxazole-4,5-diones**	-0.39	-0.21	-0.44	-0.46	-0.54	0.26

*Druglikeness scores were calculated using molinspiration chemoinformatics pharmacological database (https://www.molinspiration.com/cgi-bin/properties).

Supplementary Table 2. Bioactivity prediction (druglikeness*) of resveratrol and its derivatives for specific biological activities

Compound	GPCR ligand	Ion channel modulator	Kinase inhibitor	Nuclear receptor ligand	Protease inhibitor	Enzyme inhibitor
**Resveratrol and Methoxylated Resveratrol Derivatives**						
**Resveratrol**	-0.20	0.02	-0.20	0.01	-0.41	0.02
**Pterostilbene**	-0.13	-0.06	-0.12	0.08	-0.33	0.01
**Trimethoxystilbene**	-0.14	-0.12	-0.13	-0.02	-0.29	-0.05
**Tetramethoxystilbene**	-0.08	-0.14	-0.08	0.05	-0.22	-0.07
**Pentamethoxystilbene**	-0.07	-0.13	-0.02	-0.04	-0.17	-0.05
**Hydroxylated Resveratrol Derivatives**						
**Dihydroxystilbene**	-0.27	0.04	-0.27	-0.11	-0.47	-0.01
**Tetrahydroxystilbene**	-0.16	-0.02	-0.14	0.08	-0.36	0.04
**Hexahydroxystilbene**	-0.30	0.04	-0.19	-0.19	-0.33	0.05

*Druglikeness scores were calculated using molinspiration chemoinformatics pharmacological database(https://www.molinspiration.com/cgi-bin/properties).

Supplementary Table 3. Chemical and pharmacological properties of Lapachol, β-lapachone and derivatives.

Compound	PubChem CID	Molecular Formula	Molecular Mass (Da)	logP	tPSA (Å^2)	nrotb	Lipinski’s rule violations	Molecular volume (Å^3)	Canonical SMILES
**Lapachol**	3884	$C_{15}H_{14}O_3$	242.27	3.16	54.37	2	0	223.94	CC(=CCC1=C(C2=CC=CC=C2C(=O)C1=O)O)C
**ß-lapachone**	3885	$C_{15}H_{14}O_3$	242.27	2.95	43.38	0	0	219.96	CC1(CCC2=C(O1)C3=CC=CC=C3C(=O)C2=O)C
**α-lapachone**	72732	$C_{15}H_{14}O_3$	242.27	2.95	43.38	0	0	219.96	CC1(CCC2=C(O1)C(=O)C3=CC=CC=C3C2=O)C
**3-iodo-ß-lapachone**	N.A.	$C_{15}H_{13}IO_3$	368.17	3.43	43.38	0	0	243.97	C1=C2C(=CC=C1)C3=C(C(C2=O)=O)CC(C(C)(C)O3)I
**3-I-α-lapachone**	N.A.	$C_{15}H_{13}IO_3$	368.17	3.43	43.38	0	0	243.97	C1=C2C(=CC=C1)C(C3=C(C2=O)OC(C(C3)I)(C)C)=O
**naphtho[2,1-d]oxazole-4,5-diones**	N.A.	$C_{13}H_9NO_3$	227.22	2.16	60.17	1	0	227.22	C1(C(C3=C(C2=C1C=CC=C2)OC(=N3)CC)=O)=O

N.A. compounds not available at Pubchem database. logP values, tPSA (topological polar surface area), nrotb (rotatable bonds), Lipinski's rule violations and molecular volume were extracted from molinspiration chemoinformatics pharmacological database(https://www.molinspiration.com/cgi-bin/properties) after insertion of Canonical SMILES representation of each compound. Canonical Smiles were collected from PubChem database.

Supplementary Table 4. Chemical and pharmacological properties of resveratrol and derivatives.

Compound	PubChem CID	Molecular Formula	Molecular Weight	logP	Total polar surface área	Rotatable bonds	Rule of five violations	Molecular volume	Canonical SMILES
**Resveratrol and Resveratrol Methoxylated Derivatives**									
**Resveratrol**	445154	C14H12O3	228.24	2.99	60.68	2	0	206.92	C1=CC(=CC=C1C=CC2=CC(=CC(=C2)O)O)O
**Pterostilbene**	5281727	C16H16O3	256.3	4.06	38.70	4	0	241.98	COC1=CC(=CC(=C1)C=CC2=CC=C(C=C2)O)OC
**Trimethoxystilbene**	5388063	C17H18O3	270.32	4.59	27.70	5	0	259.51	COC1=CC=C(C=C1)C=CC2=CC(=CC(=C2)OC)OC
**Tetramethoxystilbene**	5354004	C18H20O4	300.3	4.40	36.94	6	0	285.05	COC1=CC(=C(C=C1)C=CC2=CC(=CC(=C2)OC)OC)OC
**Pentamethoxystilbene**	9818893	C19H22O5	330.4	4.17	46.17	7	0	310.60	COC1=CC(=CC(=C1)C=CC2=CC(=C(C(=C2)OC)OC)OC)OC
**Hydroxylated Resveratrol Derivatives**									
**Dihydroxystilbene**	5282363	C14H12O2	212.24	3.54	40.46	2	0	198.91	C1=CC(=CC=C1C=CC2=CC=C(C=C2)O)O
**Tetrahydroxystilbene**	5281717	C14H12O4	244.24	2.72	80.91	2	0	214.94	C1=CC(=C(C=C1O)O)C=CC2=CC(=CC(=C2)O)O
**Hexahydroxystilbene**	129837003	C14H12O6	276.24	3.37	121.37	2	1	230.98	C1=CC=C(C=C1)C=C(C2=C(C(=C(C(=C2O)O)O)O)O)O

logP values, tPSA (topological polar surface area), nrotb (rotatable bonds), Lipinski's rule violations and molecular volume were extracted from molinspiration chemoinformatics pharmacological database(https://www.molinspiration.com/cgi-bin/properties) after insertion of Canonical SMILES representation of each compound. Canonical Smiles were collected from PubChem database.
